# MicroRNA-466 inhibits the aggressive behaviors of hepatocellular carcinoma by directly targeting metadherin

**DOI:** 10.3892/or.2022.8357

**Published:** 2022-06-27

**Authors:** Chen Jia, Desheng Tang, Chen Sun, Lei Yao, Fujun Li, Yanhua Hu, Xinchen Zhang, Dequan Wu

Oncol Rep 40: 3890–3898, 2018; DOI: 10.3892/or.2018.6763

Following the publication of this paper, it was drawn to the Editors' attention by a concerned reader that certain of the flow cytometric data shown in Figs. 2C, 4C and 5C, and the cell invasion assay data shown in Figs. 2D and E, 4D and E and 5D and E were strikingly similar to data appearing in different form in other articles by different authors. In addition, the authors independently contacted the Editorial Office to request that this paper be retracted owing to the fact that they had not obtained approval from the Ethics Committee of The Second Affiliated Hospital of Harbin Medical University to use the tissue samples, even though they had written that this study was approved by the Ethics Committee, and written informed consent had been provided by all patients enrolled in the research.

Owing to the fact that the contentious data in the above article had already been published elsewhere, or were already under consideration for publication, prior to its submission to *Oncology Reports*, and the authors' own admission of serious problems concerning the ethical nature of the study, the Editor has decided that this paper should be retracted from the Journal. The authors have agreed to the decision to retract the paper. The Editor apologizes to the readership for any inconvenience caused.

